# Ocean acidification and warming will lower coral reef resilience

**DOI:** 10.1111/j.1365-2486.2010.02364.x

**Published:** 2011-05

**Authors:** Kenneth R N Anthony, Jeffrey A Maynard, Guillermo Diaz-Pulido, Peter J Mumby, Paul A Marshall, Long Cao, Ove Hoegh-Guldberg

**Affiliations:** * Global Change Institute, and ARC Centre of Excellence for Coral Reef Studies, The University of QueenslandSt Lucia, QLD 4072, Australia; † Australian Centre of Excellence for Risk Analysis, School of Botany, University of MelbourneParkville, VIC 3010, Australia; ‡ Griffith School of Environment and Australian Rivers Institute – Coasts & Estuaries, Nathan Campus, Griffith University170 Kessels Road, Nathan, QLD 4111, Australia; § Great Barrier Reef Marine Park AuthorityTownsville QLD 4810, Australia; ¶ Department of Global Ecology, Carnegie InstitutionStanford, CA 94305, USA

**Keywords:** climate change, coral reefs, herbivory, ocean acidification, resilience

## Abstract

Ocean warming and acidification from increasing levels of atmospheric CO_2_ represent major global threats to coral reefs, and are in many regions exacerbated by local-scale disturbances such as overfishing and nutrient enrichment. Our understanding of global threats and local-scale disturbances on reefs is growing, but their relative contribution to reef resilience and vulnerability in the future is unclear. Here, we analyse quantitatively how different combinations of CO_2_ and fishing pressure on herbivores will affect the ecological resilience of a simplified benthic reef community, as defined by its capacity to maintain and recover to coral-dominated states. We use a dynamic community model integrated with the growth and mortality responses for branching corals (*Acropora*) and fleshy macroalgae (*Lobophora*). We operationalize the resilience framework by parameterizing the response function for coral growth (calcification) by ocean acidification and warming, coral bleaching and mortality by warming, macroalgal mortality by herbivore grazing and macroalgal growth via nutrient loading. The model was run for changes in sea surface temperature and water chemistry predicted by the rise in atmospheric CO_2_ projected from the IPCC's fossil-fuel intensive A1FI scenario during this century. Results demonstrated that severe acidification and warming alone can lower reef resilience (via impairment of coral growth and increased coral mortality) even under high grazing intensity and low nutrients. Further, the threshold at which herbivore overfishing (reduced grazing) leads to a coral–algal phase shift was lowered by acidification and warming. These analyses support two important conclusions: Firstly, reefs already subjected to herbivore overfishing and nutrification are likely to be more vulnerable to increasing CO_2_. Secondly, under CO_2_ regimes above 450–500 ppm, management of local-scale disturbances will become critical to keeping reefs within an *Acropora*-rich domain.

## Introduction

A fundamental question in ecology is to what extent local vs. global processes drive ecosystem dynamics (Davis *et al*., 1998; Karlson & Cornell, 1998; Walther *et al*., 2002;). Coral reefs, which are highly diverse and valuable ecosystems, are under increasing threat from both global climate change and local-scale stressors (Wilkinson, 2004; Hoegh-Guldberg *et al*., 2007;). At the global scale, ocean warming is predicted to lead to an increasing frequency and intensity of coral bleaching events (Hoegh-Guldberg, 1999) and associated mortality (Anthony *et al*., 2007). Also, ocean acidification due to the uptake of CO_2_ (Sabine *et al*., 2004) is predicted to lead to reduced rates of calcification for most marine calcifying organisms including corals (Kleypas & Langdon, 2006). By reducing the growth potential and survivorship of corals, ocean warming and acidification are likely to change the competitive hierarchy of corals and macroalgae – at least indirectly by reducing the ability of corals to maintain or rapidly colonize available space following disturbances (Carilli *et al*., 2009). Generally, differential changes in the growth rates of species competing for a limited resource, such as space, influence the equilibrium abundances of the competing species (e.g. Jensen, 1987). At the local scale, overfishing of herbivores can reduce the top-down control of macroalgae (Bellwood *et al*., 2004; Mumby *et al*., 2007;) while nutrient enrichment may stimulate macroalgal growth rates (Schaffelke & Klumpp, 1998) – factors that work in combination to promote shifts towards algal dominance.

The effects of global and local-scale disturbances on coral reefs have been the focus of several reviews (e.g. Hughes *et al*., 2003; Hoegh-Guldberg *et al*., 2007;). Few studies, however, have provided formal quantitative analyses of how global impacts will interact with local stressors (but see Jackson *et al*., 2001; Halpern *et al*., 2008;). In particular, the relative roles of global and local disturbances in driving reef system resilience, as defined by the system's ability to absorb and recover from impact while retaining functional and structural integrity (Nyström *et al*., 2000; Folke *et al*., 2004;), are largely unknown. The resilience of coral reefs has been attributed mostly to the top-down control of algal biomass via grazing (Hughes, 1994; Mumby *et al*., 2007;). Bottom-up processes such as nutrient enrichment also affect coral–algal dynamics (Lapointe, 1997; McCook *et al*., 2001;), but only under reduced grazing (Mumby *et al*., 2006) – and have been considered of secondary importance in the context of phase shifts between corals and fleshy macroalgae (Hughes *et al*., 1999; Jompa & McCook, 2002;). Importantly, however, no studies have formally analysed the role of ocean warming and acidification as a factor affecting coral and macroalgal dynamics. Interactions between corals and macroalgae are implicit functions of coral and algal physiology, growth and mortality (Tanner, 1995), but have not been accounted for in their relationship to environmental change over time (Tanner *et al*., 1994; Mumby *et al*., 2007;). A recent review suggests that the equilibrial states of reef systems are sensitive to ocean acidification (Hoegh-Guldberg *et al*., 2007). However, to understand how reef-community states and their resilience to perturbations vary under changing global environmental conditions (Scheffer *et al*., 2001), the key variables ocean acidification and warming and their interactions with local-scale disturbances must be formally accounted for under nonequilibrium conditions.

In the context of reef resilience, ocean acidification, reduced grazing and nutrification can be viewed as press-type processes that define axes of changing environmental conditions, whereas warming increases the frequency of acute (stochastic) perturbations (thermal anomalies), potentially shifting the reef community to low-coral states with increasing frequency and intensity. Here, we develop a novel mechanistic resilience model and analyse how increasing atmospheric CO_2_ (forcing ocean acidification and warming) and local-scale processes (herbivory and nutrification) will interact and drive the dynamics and resilience patterns of a simplified benthic reef-community system consisting of three common groups: branching corals, fleshy macroalgae and turfs (free space for coral and algal colonization). We use established relationships between ocean chemistry and coral growth rates and between ocean warming, coral bleaching and mortality, as input functions into the resilience model for the benthic model reef community. We then run a series of simulations to address a key question: how will the resilience of this system vary under different ocean acidification, warming, and algal grazing scenarios? To answer this question we focus on determining the threshold levels of CO_2_ and grazing for when the community shifts from a coral-dominated regime (high coral resilience) to a regime of alternate coral-algal states (low coral resilience) and further to an algal-dominated regime (very low coral resilience).

## Methods

### Model development

**Fig. 1:**
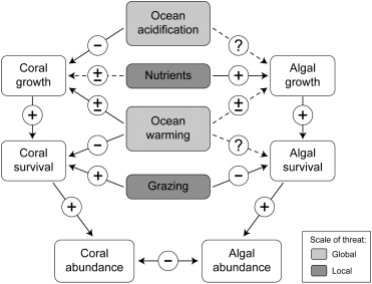
Conceptual model outlining the functional links between environmental factors, vital rates and dynamics of corals and macroalgae. Arrows indicate processes that have positive (+), negative (−) or unknown (?) effects on growth or survival. Solid arrows are primary processes that are included explicitly in the analyses whereas dashed arrows are secondary processes that are omitted from formal analyses but discussed.

We extend an existing model (Mumby *et al*., 2007) to analyse how increasing CO_2_ and the local-scale disturbances overfishing and nutrification operate mechanistically in defining coral resilience. Our analytical approach builds on a parsimonious model for the dynamics of corals and macroalgae, using growth and mortality as the vital rates. Our approach does not provide absolute measures of coral and macroalgal abundance with high confidence, but allows an analytical comparison of the relative roles of environmental and ecological processes as drivers of resilience patterns – a comparison that would otherwise be intractable using more complicated community models (see also Mumby *et al*., 2007). We use a benthic system described by three main groups, branching corals (*C*), fleshy macroalgae (*M*) and ‘free’ space (*F*, including thin algal turfs and crustose coralline algae). While this simple system does not resolve the dynamics of complex reef communities, it allows questions to be addressed regarding the effect of complex environmental scenarios on reef resilience. The system is driven by three key processes: (i) mortality of corals and macroalgae, (ii) the rate at which corals and macroalgae grow and recruit into areas of free space, and (iii) the probability of competitive success by macroalgae over corals (the factors and processes used to construct the coral reef resilience framework are outlined in Fig. 1). We focused primarily on grazing as the local driver to facilitate assessments of the relative effects of global CO_2_ on coral reefs within the context of a large body of literature on local key disturbances. To partly account for other local or regional disturbance factors such as storm damage, crown-of-thorns starfish outbreaks, ultraviolet radiation and other physical stressors that do not have a clear correlation with CO_2_, we included a significant stochastic background mortality for corals in the simulations. For analytical tractability we used the following set of four assumptions: (1) ocean acidification primarily affects coral calcification, (2) warming mainly affects coral mortality via bleaching, (3) grazing affects algal mortality only and (4) nutrients affect the growth rate of algae only. We acknowledge that each process may have multiple secondary effects and we have outlined these with dashed lines (Fig. 1). For example, warming may also lead to changes in the growth rates of macroalgae (Diaz-Pulido *et al*., 2007) and acidification may exacerbate bleaching (Anthony *et al*., 2008); or such processes may cause shifts in species composition which could modify the strength of the competitive processes and the outcomes. However, rather than accounting for secondary effects in the main analysis, we assess the potential role of such effects in separate sensitivity analyses.

We limit our analyses of coral resilience to members of the coral genus *Acropora*, which are the key source of surface reef structure, supporting a high diversity of associated reef species characteristic of the Indo-Pacific (Wallace, 1999; Veron, 2000; Bellwood & Hughes, 2001;). Also, species of *Acropora* are generally sensitive to thermal stress (Marshall & Baird, 2000; Loya *et al*., 2001;) and ocean acidification (Anthony *et al*., 2008), and thus are an important indicator group for estimating climate change and ocean acidification effects on coral reefs. For macroalgae we base our analyses on the genus *Lobophora*, which is frequently found in competition with branching *Acropora* (Diaz-Pulido *et al*., 2009).

The dynamics of corals, macroalgae and free space within 2-dimensional reef patches can be described by a set of two coupled differential equations where parameters *d*_C_ and *g*_M_ are the mortality rates of corals and macroalgae, respectively, the parameter *a* is the probability that macroalgae win over corals in space competition, and parameters *γ* and *r*_C_ are the conditional (relative) rates at which macroalgae and corals grow and recruit into areas of free space, respectively (see Table 1 for summary definitions of all variables and parameters used). All rates are constrained by the relative abundances of the interacting groups, hence 
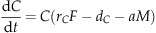
(1)
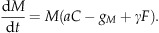
(2)

**Table 1:** Summary of symbols, functions and parameter estimates used in the model

Symbol	Unit	Interpretation	Range/function	Source
*C*	nd	Proportion of reef area covered by corals (*Acropora* sp, 2-dimensional)	0–1	–
*M*	nd	Proportion of reef area covered by macroalgae (*Lobophora* sp.)	0–1	–
*F*	nd	Proportion of reef area covered by turfs or bare substrate	0–1	–
*a*	%	Probability of competitive wins of macroalgae over corals	*w* exp(−*φ*·*C*)*w*=0.83*φ*=0.012	Mumby *et al*. (2006)
*γ*	%	Rate of colonization of free space (turfs and CCA) by fleshy macroalgae	50–90	This study
*r*_C_	%	Rate of colonization of free space by corals	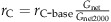	This study
*r*_C-base_	%	Baseline coral colonization rate (at 380 ppm CO_2_)	50–90	This study
*g*_M_	%	Mortality of fleshy macroalgae due to grazing (annual removal by herbivores)	30–60	Mumby *et al*. (2007)
*d*_C_	%	Annual rate of coral mortality	*M*_B_·*B*+*d*_C-base_	This study
*d*_C-base_	%	Baseline or background coral mortality (at 380 ppm CO_2_)	1–10	Tanner *et al*. (1994)
			10 ± 10	This study
Ω_arag_	nd	Aragonite saturation state	2–3.8	This study
*T*	°C	Sea surface temperature	24–32	This study
*T*_opt_	°C	Temperature at which rate of calcification for *Acropora* is maximized	27–28	This study
*κ*_T_	nd	Function for calcification response to *T*	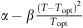	This study
*λ*	nd	Exponent for *T*-dependent calcification response to Ω_arag_	0.42 ± 0.09, *P*=0.002	This study
*α*	nd	Offset for calcification response to *T*	9.70 ± 0.68, *P*<0.001	This study
*β*	nd	Regression coefficient for calcification response to *T*	18.83 ± 5.86, *P*=0.015	This study
*G*_net_	% month^−1^	Net rate of coral calcification	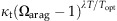	This study
*G*_rel_	nd	Ratio of projected to baseline net coral calcification	*G*_net proj_/*G*_net base_	This study
*DHM*	°C month	Size of the annual thermal anomaly (degree heating months) for *Acropora* bleaching	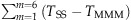	Maynard *et al*. (2008)
*DHM*_thr_	°C month	Thermal anomaly threshold for *Acropora* bleaching	0.9 ± 0.3	Maynard *et al*. (2008)
*R*_TB_	°C^−1^ month^−1^	Coefficient relating thermal anomaly to bleaching risk	25.2 ± 4.8	Maynard *et al*. (2008)
*B*	nd	Proportion of bleached *Acropora* colonies in the assemblage	R _TB·(*DHM*−*DHM*_Thr_)_	Maynard *et al*. (2008)
*d*_CB_	%	Mortality of bleached *Acropora* colonies	20–40	J.A. Maynard (unpublished data)

The term ‘nd’ indicates nondimensional (relative) units.

**Fig. 2:**
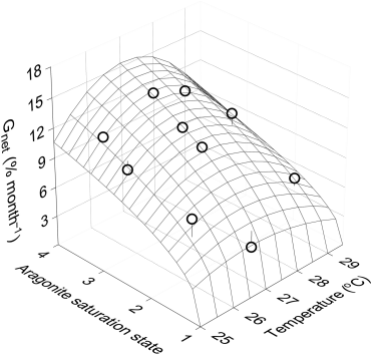
Variation in calcification responses of *Acropora intermedia* as a function of aragonite saturation state (Ω_arag_) and temperature (*T*) during 2-month experiments under varying CO_2_ dosing regimes. Data for 25.5 and 29°C are from Anthony *et al*. (2008) and data for 27°C are from Diaz-Pulido *et al*. (2010). The empirical model [Eqns (3) and (4)] explained 84% of the variation in Ω_arag_ and *T*. See Table 1 for summary results of regression analysis and estimates for parameters *λ*, *α* and *β*.

Because the sum of corals, macroalgae and free space equal unity, free space (turfs) can be described as *F*=1−*M*−*C*. Although the competitive ability of adult corals far exceed that of juveniles (Connolly & Muko, 2003), our model does not distinguish competitive strengths between juveniles and adults, potentially overestimating the capacity for coral replenishment following disturbances. Also, we consider space colonization via growth of adult colonies to occur more prominently than via larval recruitment (Connell *et al*., 1997; Diaz-Pulido *et al*., 2009;). Competitive strengths of corals are thus largely determined by the effect of the environmental variables on their rates of growth and mortality. To parameterize the model by ocean acidification and warming, we converted the constants for coral growth rate, *r*_C_, and coral mortality, *d*_C_, to functions parameterized by aragonite saturation state, Ω_arag_, and sea surface temperature, *T*. These are two of the key environmental parameters relevant to coral growth in tropical seas (Lough & Barnes, 1997; Langdon & Atkinson, 2005; Kleypas *et al*., 2006; Silverman *et al*., 2007;). The rate of coral calcification is a proxy for linear extension, which is an important mechanism of space-filling and competition (Lang & Chornesky, 1990) and data suggest that changes in calcification rate are proportional to changes in linear extension rate (Jokiel *et al*., 2008; De'ath *et al*., 2009;). To partly account for the variation in calcification responses of *Acropora* under varying aragonite saturation state and temperature we fit an empirical model for net rate of calcification (*G*_net_) to experimental growth data for *A. intermedia* on Heron Reef (southern Great Barrier Reef, Australia). We used the relative increase in buoyant weight of groups of *A. intermedia* branches (*N*=20 per treatment level) exposed for 8 weeks to three or four CO_2_ dosing regimes (affecting Ω_arag_) and two temperatures in a flow-through aquarium system (for details, see Anthony *et al*., 2008). To approximate a range of qualitatively different functional responses to Ω_arag_ and *T* (e.g. Langdon & Atkinson, 2005), we used a model of the form 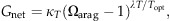
(3)where *κ*_*T*_ and *λ* are temperature-dependent functions accounting for the strength and shape of the calcification response to variation in Ω_arag_ and *T*. The temperature response of *κ*_T_ was assumed to be symmetrical around the optimal temperature for calcification (*T*_opt_, assumed to be near the maximum nonbleaching temperature of 27–28 °C at Heron Island) thus 
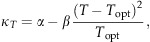
(4)where *α* and *β* are regression parameters. The fit of the model, which explained 83% of the variation in *T* and Ω_arag_ is shown in Fig. 2. The curvilinear response is consistent with the response variation reported for individual coral colonies or branches (Langdon & Atkinson, 2005). Parameter estimates are presented in Table 1.

**Fig. 3:**
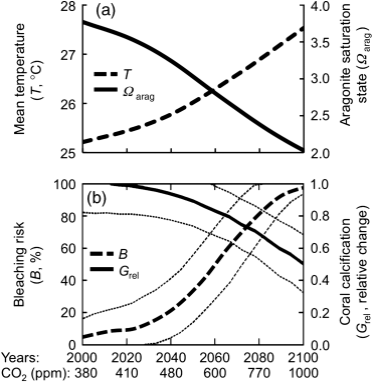
Projections of ocean warming and acidification and predicted responses by *Acropora* for the southern Great Barrier Reef, Australia. (a) Mean sea surface temperatures (*T*) and aragonite saturation states (Ω_arag_) for the A1FI carbon emission scenario for the southern Coral Sea as estimated by the UVic global carbon cycle model. (b) Projected bleaching risk (dashed lines) and projected relative change in coral calcification of *Acropora intermedia* for the 6-month period (October–March) that includes the Austral summer. Confidence bands (dotted lines) are standard deviations determined by Monte Carlo analyses.

To estimate relative changes in rates of coral growth (calcification) as a function of ocean acidification and warming, we used the baseline transition probability that corals can colonize free space, *r*_C-base_, multiplied by the ratio of projected net rates of calcification, *G*_net_, (forced by projected *Ω*_arag_ and *T*) relative to the calcification rate at the study baseline (380 ppm), *G*_net base_ (Fig. 3). Thus, 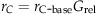
(5a)
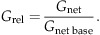
(5b)

Contrary to ocean acidification, ocean warming occurs as stochastic events (thermal anomalies), projected to increase in frequency and severity (Hoegh-Guldberg, 1999; Donner *et al*., 2009;). To accommodate such stochasticity, we express annual coral mortality risk, *d*_C_, as a function of three variables: (1) background mortality in the absence of bleaching, *d*_C-base_, (2) the amount of coral bleaching due to thermal stress, *B* (percentage of *Acropora* colonies scored as pale or white, (Maynard *et al*., 2008), which is a function of the size of the annual thermal anomaly, degree heating months (*DHM*, e.g. Eakin *et al*., 2009), and (3) the proportion of bleached corals expected to die during that year, *d*_CB_. Thus, 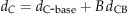
(6)
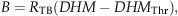
(7)where *R*_TB_ is the probability that thermal bleaching will occur and *DHM*_Thr_ is the thermal anomaly threshold for the onset of bleaching (Maynard *et al*., 2008). *DHM* for each year was calculated as the accumulated monthly exceedance of the maximum monthly mean temperature, *T*_MMM_, experienced during the previous decade 
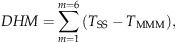
(8)where *m* is months during the heating season. See Table 1 for a summary of parameter values.

### Model simulations

We set the parameter baseline ranges for coral, *r*_C-base_, and macroalgal, *γ*, growth rates to 50–90%, representing the probability that corals (*Acropora*) or macroalgae (*Lobophora*) will colonize a free space (turfs) opened by a disturbance (Table 1). We chose similar ranges of baseline growth rates (linear horizontal extension) for both groups as this range approximates that observed for branching *Acropora* and *Lobophora* at Heron Island under herbivore exclusion (Diaz-Pulido *et al*., 2010). Also, by choosing broad ranges for *r*_C-base_ and *γ*, model projections are likely to be representative of species groups rather than individual species.

We used projected aragonite saturation state, Ω_arag_, and sea surface temperatures, *T*, for the 6-month period (October–March) that included the Austral summer for the southern Coral Sea (151.2–154.8°E, 21.6–23.4°S) as CO_2_-forced input variables into coral growth and mortality models under the fossil-fuel-intensive A1FI scenario by the IPCC (Nakicenovic *et al*., 2000). Projections of atmospheric CO_2_ and associated Ω_arag_ and *T* were generated using the University of Victoria Earth system model (UVic), which simulates the global climate and carbon cycle (Weaver *et al*., 2001; Schmittner *et al*., 2008;). To account for seasonal variation in *T*, annual means were blended with modelled seasonal variation and monthly satellite records (MODIS; oceancolor.gsfc.nasa.gov) for six sites in the region (Fig. 3). Projected sea surface temperatures were then used to estimate thermal anomalies and associated bleaching (Fig. 3) and mortality risks [Eqns (6)–(8)] as a function of CO_2_.

We set up simulations so that atmospheric CO_2_ levels (the predictor variable for Ω_arag_ and *T*) were increased in 20 increments from 380 to 1000 ppm within each of three herbivore grazing rates on macroalgae (60%, 40% and 30%) to analyse explicitly the effects and potential interactions of ocean acidification, warming and grazing on the resilience of the simplified coral reef community used here. Rather than identifying the location of community equilibria, which are unrealistic by assuming the absence of acute (stochastic) disturbances, we estimated the frequency distribution of community states (coral or macroalgal abundance) during repeated (1000 model runs) 50-year community projections within each of the 20 CO_2_ bins and for each grazing scenario. The frequency and severity of stochastic disturbances such as thermal anomalies and cyclones were included in projections by modelling the variation in bleaching-induced mortality, *d*_CB_, and background coral mortality, *d*_C-base_, as Poisson distributions with means of 30% and 10%, respectively, and standard deviations of 10% (Table 1). To determine the change in resilience patterns across simulations, we used 10 different start combinations of corals and macroalgae (ranging from 0% to 100% cover) for each CO_2_ bin. This allowed us to determine the conditions under which the system allows single or alternate stable states (Scheffer *et al*., 2001), and the extent to which the system will gravitate reversibly or irreversibly to coral or macroalgal dominance. However, because the community model [Eqns (1) and (2)] does not account for the complexity of demographic variables affecting reef patch dynamics (Mumby *et al*., 2007), we did not focus on identifying specific CO_2_ or grazing threshold values for regime shifts, but instead simply compared resilience patterns among varying CO_2_ and grazing scenarios as estimated from projected probability distributions of coral abundances.

To examine the robustness of the projected resilience patterns to changes in the values of the core vital rate parameters, (1) coral growth, *r*_C_, as influenced by acidification and temperature; (2) macroalgal growth, *γ*, from varying nutrient loading, (3) coral mortality from bleaching, *d*_CBl_, and (4) competitive strength of macroalgae over corals, *a*, we ran a series of sensitivity analyses in which coral abundance projections were generated using parameter values from either end of the range used for each core parameter (Table 1). For tractability, we here focused on comparing projected coral abundance distributions for three CO_2_ bins only (380, 540 and 1000 ppm, representing present-day, mid-century and end-century levels for the A1FI scenario), and for high (60%) and low (30%) herbivore grazing rate. All model programming was developed using matlab 7.8 (MathWorks, Natick, MA, USA).

## Results

**Fig. 4:**
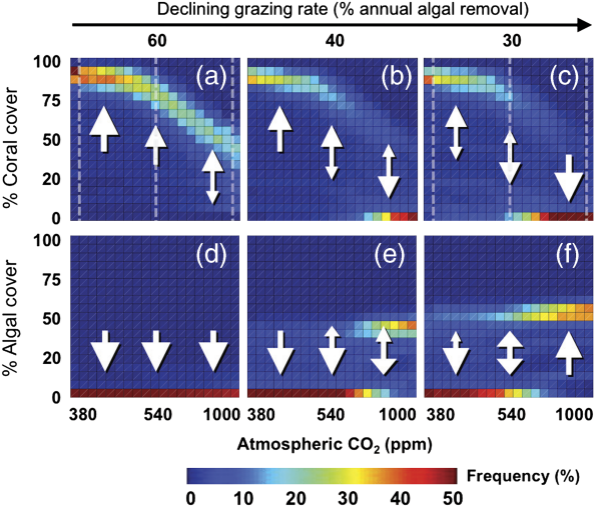
Projected frequency distribution of relative abundances of corals (*Acropora*, panels a–c) and fleshy macroalgae (*Lobophora*, panels d–f) as a function of CO_2_ forcing and herbivore grazing rate (columns, as annual mortality rate of macroalgae). Arrows indicate direction of attraction within a sequence of three regimes: coral dominance, alternate stable states (shaded areas) and algal dominance. Simulations were run using ocean acidification and grazing as press-type disturbances whereas warming and resulting coral bleaching and mortality were modelled as acute (stochastic) events with varying frequency and intensity. Dashed vertical lines indicate combinations of coral abundance projections, CO_2_ regime and grazing rates subjected to sensitivity analyses.

**Fig. 5:**
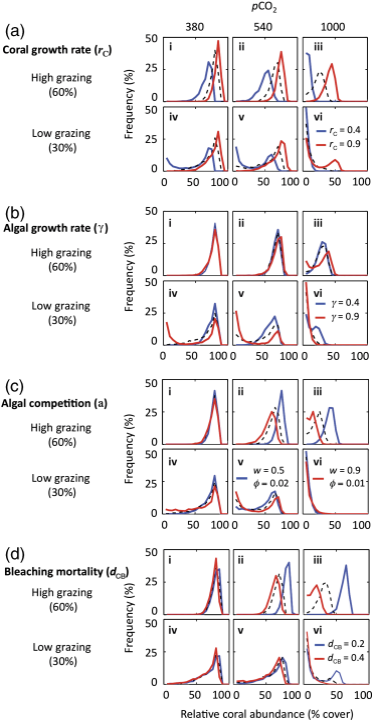
Sensitivities of coral abundance to perturbations in four core parameters (a) coral growth, (b) algal growth, (c) algal competition and (d) bleaching mortality, influencing the key vital rates of the community model, for three CO_2_ levels and two grazing rates by herbivores on macroalgae (dashed lines in Fig. 4). Each parameter was perturbed 25–30% of the set value (black dashed lines), representing lower (blue lines) and upper (red lines) bounds of the expected parameter range (Table 2). The black, dashed lines correspond to the projected coral abundance distributions for low, medium and high CO_2_ levels in Fig. 4a and c.

Our analyses of the *Acropora*/*Lobophora*/turf system indicate that ocean acidification and warming are critical drivers of change in coral resilience via impacts on coral growth rates and survivorship. For this model community system, reefs characterized by *Acropora* assemblages with low background mortality (Table 1) and high grazing rates (60% annual removal) on fleshy macroalgae (*Lobophora*), mean coral abundance was projected to fall by more than 50% by the highest CO_2_ level (1000 ppm, Fig. 4a). Interestingly, the predicted loss of corals did not lead to an increase in macroalgae (Fig. 5a), but instead an increase in free space (turf areas, not shown). Importantly, Figs 4 and 5 provide a direct view of resilience patterns as they are probability distributions (based on Monte Carlo simulations) of nonequilibrium coral and macroalgal abundances affected by press factors (grazing, acidification, nutrients) as well as disturbance frequency and intensity (bleaching and background disturbances such as cyclones).

**Table 2:** Simulation scenarios and sets of parameter ranges used in sensitivity analyses to test robustness of community projections under varying parameter values for the four core vital rates of the model

A: Scenarios	Key variables	Values
Warming and acidification	CO_2_	380, 540, 1000 ppm
Overfishing	Herbivore grazing rate	30, 60%

B: Vital rates	Parameters	Values (%)
Coral growth	*r*_C-base_	50, 90 (mean ± 30)
Algal growth	*γ*	50, 90 (mean ± 30)
Coral mortality from bleaching	*d*_*CB*_	20, 40 (mean ± 30)
Macroalgal wins over corals (coefficient in function *a*)	*w*	60, 100 (mean ± 25)
Macroalgal wins over corals (exponent in function *a*)	*φ*	0.01, 0.015 (mean ± 25)

See Table 1 for descriptions of parameters and variables.

A key result of our analyses was that warming and acidification interact with reduced grazing rates (due to overfishing or disease of herbivores) in the decline of coral resilience (i.e. referring to the probability patterns of coral abundance). Specifically, increasing CO_2_ lowered the overfishing threshold leading to shifts between (1) coral-dominance (high coral resilience), (2) alternate coral–algal states (medium coral resilience) and (3) algal-dominance (very low coral resilience). Reducing grazing rates from 60% to 40% (e.g. simulating increased fishing of herbivores) lead to a decline in coral resilience in response to increasing CO_2_ (Fig. 4b). Specifically, CO_2_ levels above around 600 ppm incurred a regime shift to alternate coral–algal states and lead to macroalgal dominance at the highest CO_2_ level (Figs 4b and 5b). Further lowering of the grazing rate to 30% exceeded the coral resilience threshold for any coral dominance above 400 ppm CO_2_ (Fig. 4c and f). The combination of high CO_2_ (700–1000 ppm) and low grazing (30%) led to a shift characterized by near complete coral loss and an increase in macroalgal cover of up to 50% (Fig. 4c and f). Thus, for the modelled system, moderate to high herbivore grazing intensity can prevent losses in coral resilience under intermediate CO_2_, but maximum grazing rates are required to maintain coral-dominated states under the very high CO_2_ levels representative of the end point of the A1FI scenario.

The patterns of model sensitivity (Fig. 5) to large (25–30%) perturbations in the values of the four core parameters (baseline coral growth, *r*_C_; algal growth, *γ*; algal competition strength, *a*; and mortality risk of corals to thermal bleaching, *d*_CB_), indicated that coral resilience patterns in Fig. 4 were generally robust to parameter variation for most combinations of CO_2_ and grazing. Detailed analyses, however, indicated that coral abundance projections had different sensitivities to different parameters, depending on CO_2_ and grazing scenario. Interestingly, whereas coral abundance projections were sensitive to coral growth for all combinations of CO_2_ and grazing, they were only sensitive to the other three parameters for some combinations of CO_2_ and grazing. Specifically, perturbing *r*_C-base_ by 30% to either side of its mean value shifted the mode of the coral abundance peaks by 5–20% along the coral cover axis for most CO_2_ and grazing scenarios. As coral growth rate was assumed to be driven by water carbon chemistry and temperature regime only, this sensitivity partly represented uncertainty in the effects of acidification and warming. However, sensitivity to coral growth variation was highest at high grazing and high CO_2_ where corals were predicted to dominate, although at low abundance (Fig. 5aiii). Coral abundance projections were insensitive to changes in algal growth rate and algal competition strength for low and intermediate CO_2_ at high grazing (Fig. 5a and b) where coral growth rates are high and algal biomass is controlled by herbivore grazing. At high CO_2_, however, a high algal growth rate (90% transition probability from turfs to macroalgae) was predicted to promote a shift from coral dominance to a regime of alternate coral and algal states (red line in Fig. 5biii). Conversely, for the low grazing scenarios, coral abundance projections were sensitive to variation in algal growth rate indicating proximity to a phase-shift threshold. Specifically, a high algal growth rate promoted regime shifts to alternate coral–algal states at low and intermediate CO_2_ (red lines in Fig. 5biv and v), and shifts from alternate coral–algal states to complete algal dominance under high CO_2_ (Fig. 5bvi). This indicates that bottom-up processes affecting algal productivity (e.g. nutrient enrichment and elevated temperature) become increasingly important as the top-down control of macroalgal biomass is reduced and CO_2_ is increased, impairing coral growth and survivorship.

Interestingly, coral abundance projections were only highly sensitive to variation in the algal competition function, *a*, for intermediate and high CO_2_ under high grazing (Fig. 5cii & iii). This is partly due to the lower survivorship and growth rate of corals in these CO_2_ regimes. At low grazing and intermediate CO_2_, an increase in algal competition strength (red line in Fig. 5civ) enhanced the tendency for alternate coral–algal states to be formed.

Variation in mortality risk of bleached coral, *d*_CB_, strongly affected the location of modes of coral abundance projections under high CO_2_, in particular at high grazing where corals were predicted to dominate (Fig. 5diii). Here, low mortality of bleached corals (20%) meant that coral abundance projections remained comparatively high (around 40% frequency of 60–70% cover) for all CO_2_ levels at high grazing. However, 20% mortality of bleached corals is likely to be unrealistically low under a high CO_2_ scenario and severe bleaching risk. Using a high mortality rate of bleached corals (40%) incurred a drop in coral abundance to below 20% cover. Under low grazing and high CO_2_, variation in bleaching mortality determined the extent to which coral abundance projections formed alternate coral-algal states or led to coral loss and algal dominance (panel vi in Fig. 5d).

## Discussion

Overfishing and/or mass mortality of herbivores (Hughes, 1994) are considered to be major drivers of community phase shifts on coral reefs (Nyström *et al*., 2000; Bellwood *et al*., 2004; Mumby *et al*., 2007;). Although increasing atmospheric CO_2_ levels is recognized as a growing threat to coral reefs worldwide (Kleypas *et al*., 2006; Hoegh-Guldberg *et al*., 2007;), the extent to which the consequent ocean acidification and warming will affect the resilience and dynamics of reef communities via interaction with overfishing forms a critical knowledge gap. Here we demonstrate, using a probabilistic resilience model building on the dynamics of a species pair of corals (*Acropora*) and fleshy macroalgae (*Lobophora*), that the effects of ocean acidification and warming on coral growth and mortality will have important impacts on coral reef resilience under increasing CO_2_. Specifically, by reducing coral growth (due to acidification) and survivorship (due to warming), increasing CO_2_ will lower the threshold value at which local and regional processes like herbivore overfishing and nutrification drive the study community from predominantly coral-dominated to predominantly algal-dominated states. Therefore, warming, acidification, overfishing and nutrification all drive the dynamics of the system in the same direction, suggesting that reduced coral resilience in a high-CO_2_ world is likely to be a consequence of both global threats and local-scale disturbances.

These findings have far-reaching implications for the health of coral reefs in the future for at least two reasons. Firstly, under a fossil-fuel intensive carbon emission path (the A1FI scenario by the IPCC) – the current global trajectory – acidification effects on coral calcification and increased coral mortality from bleaching may potentially reduce *Acropora* abundance to less than half the current abundances despite high rates of grazing and low levels of nutrification. This indicates that management efforts to maintain healthy herbivore populations and high water quality will become increasingly critical as atmospheric CO_2_ levels rise – and that such efforts could be futile in the longer-term if efforts to curb emissions growth are unsuccessful. Thus, even coral reef systems with effective management systems in place such as the Great Barrier Reef (Fernandes *et al*., 2005) and Bonaire (Burke & Maidens, 2004) are predicted to suffer increased damage from high-carbon scenarios. Secondly, and in contrast, sensitivity analyses presented here suggest that under a low CO_2_ scenario (e.g. below 540 ppm) local management effective in maintaining or restoring healthy herbivore populations (high grazing) and low nutrients can increase the chances that reefs will remain coral-dominated. Based on the likely shift from coral dominance to algal dominance under the high CO_2_ and low grazing scenario in Fig. 4c, it can be inferred that coral reefs in developing nations, where most of the world's reefs occur and overfishing and nutrification remain key concerns (Silvestre & Pauly, 1997; McClanahan, 1999; Jackson *et al*., 2001; Knowlton & Jackson, 2008;), are particularly vulnerable to acidification and warming. While coral dominance is possible in our model projections under high CO_2_ and high grazing, the combination of high CO_2_ and low grazing (herbivore loss) leads to severe coral loss even under the highly conservative assumptions made here.

The results of this study generally support the conclusions of recent reviews (e.g. Hoegh-Guldberg *et al*., 2007) that increasing CO_2_ will exacerbate effects of overfishing and nutrification on reef ecosystems. However, by formally accounting for interactions between CO_2_ effects and local-scale processes on reef state probability under both pulse and press disturbance regimes, our model analyses provide novel quantitative estimates of resilience patterns and allow assessments of the relative contribution from environmental and ecological processes in maintaining resilience. Although our projections of *Acropora* abundance as a function of CO_2_ level do not constitute accurate predictions, they reflect the relative impact of various disturbances on a simplified reef community.

It is likely that our projections of coral abundance are overestimates, given that our analyses are based on a set of conservative assumptions with respect to effects of acidification and warming on corals and macroalgae. Firstly, we consider ocean acidification to reduce the growth rate of corals (parameter *r*_C_) and assume that the growth rate of macroalgae (parameter *γ*) of the type used here (fleshy brown *Lobophora* sp.) will not be affected by acidification or warming. Results of recent experiments, however, indicate that the rate of linear extension of *Lobophora* is 20–40% enhanced under intermediate CO_2_ levels (560–700 ppm; but declined under higher CO_2_ concentrations, Diaz-Pulido *et al*., 2010). Secondly, we assume that the probability of competitive wins of macroalgae over corals (parameter *a*) will remain unchanged under varying CO_2_. Again, recent experiments indicate that ocean acidification strongly shifts the competitive coral–algal interaction in favour of the macroalgae (Diaz-Pulido *et al*., 2010), suggesting that *a* will increase with ocean acidification. Thirdly, our model assumes that rates of coral calcification are unaffected by bleaching, which is likely to overestimate coral growth rates under high CO_2_ levels where corals are likely to bleach (Carilli *et al*., 2009). Lastly, we do not formally consider effects of ocean acidification on the mortality risk of corals, thereby disregarding a likely lowering of the break resistance of corals during storms (Madin *et al*., 2008). Additionally, a recent study proposes that elevated nutrients can lower bleaching thresholds (Wooldridge & Done, 2009), suggesting that our model will underestimate bleaching-induced mortality in coastal regions.

Given the conservative assumptions listed here, the results of our model projections strongly suggest that the increase in atmospheric CO_2_ (ocean warming and acidification) and local-scale disturbances (overfishing and nutrification) will both contribute to reducing the resilience of corals this century. Also, our findings that the abundance of corals under high CO_2_ is sensitive to variation in coral growth and mortality parameters as well as to variation in algal growth and competition parameters indicate that coral resilience patterns are driven by both CO_2_ (impacting mainly corals in our model via acidification and bleaching) and nutrification (via algal growth). The increased sensitivity of the model projections for corals to variation in algal growth under low grazing (herbivore overfishing) indicates that nutrification effects on coral resilience becomes particularly important in the proximity of threshold points for grazers control of algal biomass (Mumby *et al*., 2006). Further, the relatively low sensitivity of the model to parameter variation at high CO_2_ and low grazing suggests that algal-dominated and coral-depauperate systems are persistent. While the difficulty in reversing shifts from coral- to algal-dominance has been long known (Hughes, 1994; Mumby *et al*., 2007;), the results of our analyses emphasize the risk of further increasing the persistence of undesirable reef states in a high-CO_2_ world.

In conclusion, our quantitative analyses of resilience patterns for a simplified model system bring into focus two core principles regarding the future of coral reefs under increasing CO_2_. Firstly, a failure to rapidly stabilize and reduce the concentration of CO_2_ in the Earth's atmosphere is likely to lead to significant loss of key framework builders such as *Acropora*, irrespective of the effectiveness of local management. Secondly, local reef management efforts to maintain high herbivore grazing and low nutrients have the potential to play a critical role in maintaining coral resilience while CO_2_ concentrations are stabilized.
